# Food allergy safety: a descriptive report of changing policy in a single large medical center

**DOI:** 10.1186/s13584-021-00466-w

**Published:** 2021-05-03

**Authors:** Rivki Harari, Orly Toren, Yuval Tal, Tair Ben-Porat

**Affiliations:** 1 Department of Diet and Nutrition, Hadassah-Hebrew University Medical Center, Jerusalem, Israel; 2 Department of Patient Safety and Risk Management, Hadassah-Hebrew University Medical Center, Jerusalem, Israel; 3 Allergy and Clinical Immunology Unit, Department of Internal Medicine, Hadassah-Hebrew University Medical Center, Jerusalem, Israel; 4 Department of Human Metabolism and Nutrition, Braun School of Public Health, Hebrew University, Jerusalem, Israel

**Keywords:** Food allergy, Hospital foodservice, Safety policy

## Abstract

**Background:**

Food allergy can result in life-threatening anaphylaxis and is considered an increasing public health burden. Hospitalized patients are dependent on the hospital menu to meet their nutritional needs; thus, errors in the meals provided can have a substantial impact on patients’ health outcomes. In Israel, no specific policy protocol exists to ensure food allergy safety in the setting of a hospital foodservice system.

**Objectives:**

This paper has two aims: 1) to provide an in-depth review of food allergy as a major public health concern and 2) to report actions taken in a single large medical center, as an ongoing project that aimed to ensure patients’ safety, and which ended in developing policy on this matter.

**Results:**

During the years 2017–2019, we initiated several interventions with the goal of achieving food allergy safety and ensuring quality of care for patients with food allergies at Hadassah Hebrew University Medical Center. These included integrating food management safety into the computerized foodservice system, highlighting labels on patients’ food trays, introducing safety checks into the process of food delivery to hospitalized patients; and ensuring the nutritional requirements of patients with allergy restrictions. Moreover, changes were made in specialized menus for patients with various types of food allergy, and specific procedures were implemented regarding enteral feeding, to prevent accidental allergen exposure. All the procedures were incorporated into a written protocol that applies to all hospital employees, and the staff received the relevant training.

**Conclusions:**

Our experience suggests that methods for food allergy safety should be promoted, and that an established policy and suitable set of guidelines on this matter is required. This clearly mandates collaboration between the various sectors of the hospital, including management and the computer department; and the medical, nursing, dietetics and kitchen staffs. Furthermore, routine ongoing knowledge training programs for medical teams and kitchen staff are crucial for such implementational changes. In a technological world, computerized systems delivering food to hospitalized patients must be adapted such as to create a uniformly safe food environment of healthcare systems, and developing a suitable policy should be prioritized accordingly by hospitals across Israel, with collaboration and synergy between institutions management and the departments of nutrition and patient safety and risk management.

## Introduction

Food allergy is defined as an adverse reaction to food, mediated by an immunologic mechanism that involves specific IgE (IgE-mediated) or cell-mediated mechanisms (non-IgE-mediated) [1]. Nine food groups are considered to be major allergens: milk, egg, peanuts, tree nuts, sesame seeds, wheat, soy, fish and crustacean shellfish [2–4]. Food allergy can result in life-threatening anaphylaxis [1], and impose a personal and public health burden, which can impair quality of life, and increase health-related costs and service demands [5]. Clinical management includes short-term interventions to manage acute reactions, and long-term strategies, primarily by means of dietary modifications [1], to minimize risks of further reactions. The prevalence of food allergy has been increasing in recent decades, particularly in westernized countries [5], and is considered an increasing public health concern, affecting millions of persons worldwide [6]. Of particular worry is the apparent acceleration in prevalence of food allergy in older children and teenagers, ages for which the risk of death due to food anaphylaxis is the highest [5]. Novel therapeutic approaches including immunological tolerance are in process and may hold great potential. Nevertheless, for individuals who are already diagnosed, complete avoidance is still the main established method for preventing a reaction. This is not easily achieved, particularly when effective policies and practices are not implemented in places where foods are purchased or consumed, such as hospitality and food service industries [2]. Furthermore, even with the most stringent management practices, accidents, such as cross-contact events, can occur. Thus, many stakeholders, including policy makers, the food industry, scientists, clinicians, and especially individuals with food allergy and their caregivers, are concerned about the constant life-threatening miscalculations or mistakes that may occur, and the lack of effective treatment and clear approaches for preventing food allergy events [2]. Specifically, patients requiring therapeutic diets in a hospital setting are at risk of exposure to dietary errors that may pose an acute threat to their safety [7]. Meal-related errors among patients prescribed therapeutic (specialized) diets may pose chronic and acute health problems. Thus, the provision of accurate therapeutic meals remains an important aspect of a patient’s immediate safety, especially for individuals with food allergy [7, 8].

A paucity of policy on intervention and protocols relating to food allergy safety in the hospital setting has been described worldwide [9–11]. Currently, no specific policy protocol exists in Israel to ensure food allergy safety in the setting of a hospital foodservice system.

Following a near-miss event of anaphylaxis allergic reaction due to food cross-contact in our hospital, the Department of Nutrition together with the Department of Patient Safety and Risk Management initiated several safety-related interventions during the years 2017–2019. These were aimed to decrease the risk of food allergy events and to promote relevant policy at the Hadassah Hebrew University Medical Center, Ein Kerem campus. The process included several steps that involved changes in practices and policies through various sectors of the hospital. The current report is aimed to summarize the rationale and details of actions that were taken at our center, and to discuss possible implications on future policy, for establishing food allergy safety protocols in other hospitals and/or a national suitable policy for the hospital setting. Furthermore, we review the existing literature on food allergy as a major public health concern, and specifically in the setting of health care systems.

## Food allergies: causes, treatment and prevention

### Characterization of food allergies

Several studies have described increasing prevalence of anaphylaxis. Food is the most common cause of this reaction, accounting for up to 30% of fatalities [12]. Food allergy prevalence has been reported in the range of 4–20% [6, 13–15], and is generally higher in children than in adults [1]. However, determining the actual prevalence of food allergy remains elusive, due to its diverse manifestations and variable severities, and the methodological differences between studies. The latter include differences in definitions of allergy, in the populations and the specific foods evaluated, and in study designs [6]. Furthermore, self-reported food allergy rates are substantially higher than those confirmed by medically supervised oral food challenges [6, 15]. Nevertheless, many health care experts agree that a real increase in food allergy has occurred and that it is unlikely to be entirely due to an increase in awareness and better diagnostic tools [2]. Factors that have been speculated to contribute to the elevated prevalence of allergic disease include changing practices in food manufacturing (e.g., alterations in the production of processed foods), decreases in microbial exposure early in life, and the changing microbiome [16].

### Types of food allergies and the source of exposure

Allergies are distinguishable from other adverse reactions to foods in that they involve an immune response. Thus, for example, intolerance (e.g., lactose intolerance) is not considered a food allergy [6]. A rather short list of foods account for most of the more serious food allergy burden, namely peanuts, tree nuts, sesame seeds, fish, shellfish, eggs, milk, wheat and soy [6]. The most common foods causing allergy in children are peanuts (32.0%), tree nuts (22.7%), milk (17.2%) and eggs (16.4%). In contrast, among adults, the most common foods reported are shellfish (34.4%), tree nuts (20.0%) and peanuts (12.2%) [12, 17]. Among young adults in Israel, the most common food allergens are reported as tree nuts, cow’s milk, peanuts, fish, sesame and eggs [15]. Although reports in the literature show that most anaphylaxis cases are associated with peanuts and tree nuts, in Israel most anaphylaxis cases are caused by cow’s milk, tree nuts and sesame food allergy [3, 18]. Along this line, early introduction of peanuts in infants at high risk for peanut allergy was shown to prevent peanut allergy [19–21]. Thus, recent guidelines for the prevention of peanut allergy have recommended early introduction of peanut-containing foods into the diets of infants at various risk levels for peanut allergy [22]. Generally, the order of prevalence of specific allergens varies in different countries, probably due to an interaction of genetic factors, dietary patterns, and exposure to new allergenic products early in life [23, 24]. For example, peanut allergy is very common in the UK, France, Switzerland, and North America, but very rare in Italy and Singapore [23, 24]. In Israel, peanut allergy is less common than in North America and Europe, while on the contrary, sesame allergy is more prevalent [23, 24]. Accidental exposure to food allergens can lead to significant morbidity and mortality. Exposures in restaurants and to take-out food account for a large proportion of severe reactions [25, 26]. Unexpected reactions have mostly been reported in restaurants (21–31%), at school or work (13–23%), at friends’ houses (12–35%) and at home (26–37%) [26]. Surprisingly, information on allergic reactions derived from cross-contact errors among hospitalized patients is hardly described in the literature [27]. Nevertheless, the majority of anaphylaxis deaths eventually occur in hospitals. This, together with the relatively high proportion of the population with food allergies and the decreased health status of hospitalized patients, solidifies the importance of ensuring food allergy safety in the hospital setting [9].

### Food allergy management: prevention and therapeutic approaches

Since no cure for food allergies is available, several effective and systematic steps are needed to prevent accidental allergen exposure and the potentially severe outcomes of such [6]. Food-related anaphylaxis is primarily diagnosed by signs and symptoms, and supported by the identification and confirmation of a culprit food allergen [12]. The most important step in diagnosing a food allergy is obtaining a thorough medical history, which includes the type of food ingested, the type of symptoms and the timing of the reaction [16]. Anaphylaxis is defined as a serious allergic reaction that is rapid in onset and may cause death [12]. First-line treatment of anaphylaxis is intramuscular administration of epinephrine; treatment should be provided even if the diagnosis is uncertain, since there are no absolute contraindications to the use of epinephrine [12]. Long-term management generally focuses on dietary interventions and dietary avoidance, which results in complete or almost complete resolution of symptoms [1, 12]. Dietary restrictions should eliminate the culprit food allergen(s) and be tailored to the individual’s specific allergic and nutritional needs. Thus, ideally, the patient should receive proper counseling by a registered dietitian with specific competence in food allergy [1]. Education is the key pillar of an effective long-term elimination diet: patients, their families, close relatives and caregivers should be aware of risk situations and should be instructed in acute management situations as well as in reading labels and how to avoid the relevant food allergens both in and outside the home [1]. Regarding schools, legislation regarding food allergy, dissemination of relevant information and ensuring stock epinephrine are important [6]. While early introduction of peanuts to high-risk infants is backed by convincing data [6], the effectiveness of such approach is less certain regarding other food allergies, based both on the current evidence and on recognition of the paucity of data [6]. Importantly, new prevention models and innovative therapeutic strategies aimed at a personalized approach to the patient affected by food allergy are emerging, based on the concept of immunological tolerance [28, 29]. Ultimately, such novel therapeutic approaches to food allergy may offer a reduction in the risk of allergic reactions with an ultimate goal of allowing avoided food to be fully reintroduced into the diet [28, 29].

## Approaches for establishing food allergy safety in food service systems

### Managing food allergies in food services

For an individual with a food allergy, the persistent avoidance of allergenic foods may be complex and challenging, since direct interactions with foods can occur in many public settings including foodservice systems [2]. Individuals with food allergies must depend on personnel in food service establishments to obtain allergen-safe foods, and errors can be tragic [2]. Restaurants were reported as the setting of 21–31% of unexpected allergic reactions [26]. Such errors may occur from communication problems with the consumer and from a variety of circumstances in the establishment, such as unawareness to hidden ingredients and cross-contact between foods [2]. Potential problems that have been described in regard to understanding and managing food allergy on the part of restaurants and food services include a high rate of personnel turnover, which challenges educational training programs; and low comprehension and interest in training programs on food allergy among staff members [30, 31]. The Food and Drug Administration issued a code for uniform systems and practices for food allergen safety in food service. The recommendations include: ensuring proper training in food allergy awareness, and instilling knowledge in identifying major food allergens and in symptoms of food allergy reactions [2, 32]. This code indicates the need for developing and implementing operational-specific training programs for food employees, and addressing the need of specific procedures and activities (e.g., general cleaning, managing raw foods, etc.) [2, 32]. Food allergy training programs for employees can include defining food allergens, recognizing symptoms, proper communication, special dietary requests, dealing with emergencies, the importance of food labels and proper food preparation [2, 32, 33].

### Hospital foodservice systems

Hospital foodservice is considered one of the most complicated systems in hospitals. The many interrelated factors include menus that are based primarily on clinical needs and patients’ preferences, as well as on variety, quality, aesthetics and the taste of the food [7, 34]. A therapeutic diet is prescribed to meet a medical or special nutritional need; and can constitute a part, or even the main part of a clinical treatment. Adherence to such diet may be essential for preventing immediate or long-term clinical damage [34]. Difficulties of the kitchen and the entities that link hospital food service to the medical departments (e.g., miscommunication between the medical departments to the kitchen staff due to other sectors involved in the relevant stages of patients’ care within the hospital and mostly computerized procedures involved in this process, lacking guidance to kitchen staff, etc.) can impair implementation of the nutrition program. The consequence is patients not receiving the optimal food for their needs, according to the medical and nutrition guidelines issued in the department, even if the correct nutritional instructions were recorded [35]. Food safety is a critical factor in the preparation and serving of food to hospitalized patients, who are more likely than the general population to be susceptible to foodborne illness, due to their decreased health status [7, 34]. The provision and consumption of inappropriate food or fluids by patients who require a therapeutic diet can interfere with medical treatment, and under certain circumstances, pose a risk to immediate health; this is specifically true in the case of food allergic reactions [7]. Despite this extensive list of potential consequences, only a limited number of studies have investigated the accuracy of therapeutic dietary provision in medical facilities. Moreover, none of these specifically addressed food allergy safety [7, 36, 37]. Generally, strategies that were previously demonstrated to improve meal tray accuracy in hospitals include a standardized menu formatting and spoken menu systems [38]. According to the Nutrition Division at the Ministry of Health in Israel, the continuity of care between hospital departments and hospital food services, as reflected in the serving of food that is appropriate to patients’ medical needs, is incomplete and even nonexistent in some hospital departments [35].

### Hospital food anaphylaxis prevention

Overall, we identified a paucity of documented standards of practice for the current project, as only a few hospital food anaphylaxis protocols have been published. Protocols that have been made publicly available do not always address strategies to ensure proper communication, food preparation, labeling, and delivery of the hospital menu in the context of food allergy safety [9–11]. A case in point was apparent in the description of an initiative to increase the safety of children with food allergies who were admitted to a children’s hospitals in the US [10]. The authors stated that other hospitals that they contacted reported not having a relevant protocol, and that they were handling each patient on a case by case basis. Nevertheless, two published protocols addressing this issue were identified [9, 11]. In the protocol of a public medical center in Turkey, precautions taken by the hospital kitchen included the designation of a separate section of the hospital kitchen for the preparation of food for patients with allergies, and the administration of an education program to the hospital kitchen staff [9]. In a health care food service setting in Australia, resources to help healthcare facilities safely manage patients with food allergies were integrated into a complete resource kit for food allergen management [11]. These were incorporated to create national policy practice guidelines, while addressing several aspects of food service practices. Such practices included risk management and audit verification at different stages of the meal delivery process within the hospital, from patient admission, to meal preparation and delivery, accompanied by support programs of employee training, communication and awareness.

## The project

### The intervention: methods and strategy

**Fig. 1:**
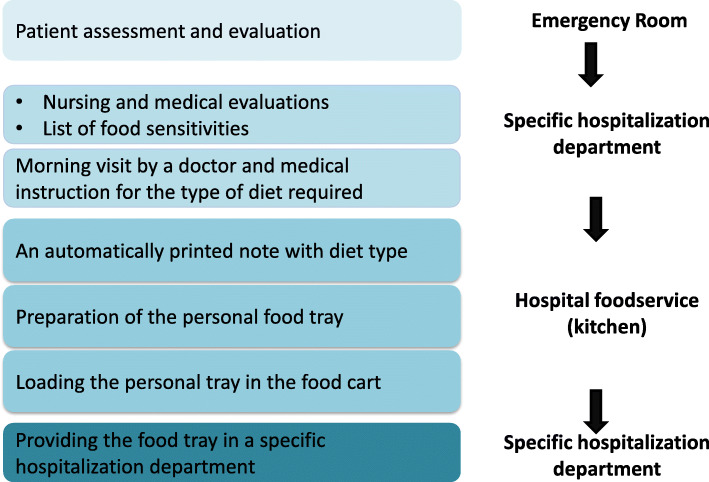
A flow chart of the intervention model implemented across several hospital sectors, to ensure food allergy safety

Our goal was to establish a protocol and policy regarding the safety of patients with food allergy in our hospital. Our main activity was to target the specific hospital processes and sectors that are involved in the relevant stages of patients’ care. These stages start with patient admission and the detection of a food allergy; and continue to general hospital processes of food preparation, delivery and serving the correct food tray to a patient. For each point and or sector involved, we aimed to address the specific actions needed for preventing an allergic reaction due to a food-triggering cause. We strived, concurrently, to ensure patients’ nutritional requirements, despite the dietary restrictions imposed by food allergies. Figure 1 presents the flow of the procedures we implemented during 2017–2019, and the relevant hospital sectors. The specific actions and strategies implemented included the following (Table 1**)**:

- ***Patient admissions:*** The protocol of patients’ admission was changed to include mandatory questions related to detecting allergies (or potential allergies) to food. Allergen status was documented in patients’ files.
- ***Management safety through the hospital computerized foodservice system:*** A separate list of the nine most common food allergens was implemented in the computerized system of the patients’ records. To emphasize an existing allergy, a specific color on the computerized record was highlighted. Additionally, feeding formula ingredients and safe enteral feeding options were reviewed to establish measures for preventing accidental allergen exposure from such sources. Computerized procedures were assimilated into the system such that the indication of a food allergy automatically blocked prescribing enteral feeding.
- ***Separate food preparation and delivery pathways for patients with food allergies:*** The ingredients of all meals were reviewed, according to course and serving. Specific lists of ingredients that are prohibited for each of the common allergies were prepared. Since breakfast and dinner are prepared at the hospital’s kitchen, a separate production line was built to prepare these meals for patients with allergies. This entailed close monitoring of meal preparation and the exclusive use of non-allergic ingredients. Lunch meals are brought in sealed food trays from an outside factory that has a high standard for non-allergen food production. Additionally, for all patients with food allergies, a diet label is generated, which includes the patient’s details, a highlighted title of “Allergy Diet by Sensitivity” and the type of food allergy. Routine rounds to ensure the safety of all the food preparation and delivery processes were implemented as functions of the kitchen management and of a dedicated dietitian from the dietary service.
- ***Staff education program:*** Food allergy awareness and process training is included in the orientation of all staff members who are involved in the process, including physician and nurses, ward administrators and food service staff.
- ***Food allergy protocol -*** A unique protocol that applies to the entire hospital was written. The protocol includes all the safety measures to be implemented for patients’ identification and documentation, and for relaying the information on food allergies to the appropriate functionaries: food preparation, food delivery, training of various hospital staff, management monitoring and reporting critical incidents. This protocol was also delivered through training given to different hospital staff employees (i.e., employees that are involved in clinical admission and in the process of food delivery to patients) as well as presented in a few national conferences.
- ***Monitoring critical/near miss events:*** A process to record, monitor and evaluate critical events /near misses was implemented. Accordingly, such events are to be reported and evaluated at meetings of the Quality and Safety Department. When conclusions are relevant to the general hospital, specific steps should be taken and implemented.

**Table 1:** Actions and strategies implemented to achieve food allergy safety in Hadassah Medical Center

Aim and scope of the intervention	Target workstations	Hospital sectors/ employees involved	New practice implemented	Practice prior to intervention
Getting the intervention approved	Hospital administrators	Hospital director, computer division, risk management dept., quality and safety dept	Approval of the intervention and prioritization of developments in the computerized system	–
Proper communication: Food allergy documentation	Computerized system	Physician / nurse during patients’ evaluation/ reception	A dedicated list of food allergens: the 9 most common and “other”	A long list of materials (including food, latex).
Proper communication: Writing instruction for patients’ food trays	Computerized system	Physician / clinical dietitian	Cancellation of “regular diet” as the default diet. Physicians prescribe diet type. To the option, “Allergy Diet by Sensitivity”, additional dietary restrictions may be added (e.g., diabetes diet).	Default diet was “regular diet”
Proper communication: Enteral feeding instruction	Computerized system	Physician / clinical dietitian	If a food allergy is indicated, an enteral feeding cannot be prescribed.	Enteral feeding instruction for patients with food allergy was possible
Proper communication and proper food preparation: Notes that label diets in the hospital kitchen	Computerized system	Computer division, kitchen employees	Notes include patients’ details; “Allergy Diet by Sensitivity” and the specified food allergy type are highlighted	The food allergy type was not highlighted
Proper communication: Daily summary report	Computerized system, hospital kitchen	Food service dietitian, Kitchen management	Daily report of all patients with food allergy in the hospital	–
Proper communication, and food labeling and delivery to patients: Highlighting food trays with food allergy diets	Hospital kitchen	Kitchen food-distribution manager, Kitchen shift manager	Highlighted placemats for food trays of “Allergy Diet by Sensitivity”	–
Proper communication and food labeling and delivery to patients: Food tray checkup	Hospital kitchen	Food service dietitian, Kitchen shift manager	Food tray checkup prior to delivery to patients	–
Defining and identifying food allergens and proper communication: Creating a list with food allergy information	Hospital kitchen, Dept. of Nutrition and Diet	Kitchen shift manager, Kitchen employees	A list of foods that cannot be integrated into allergy food trays according to allergy type and meal type, hung on a kitchen billboard	–
Proper cleaning and hygiene methods, proper food preparation	Hospital kitchen	Food service dietitian, storekeeper	Sealed food trays delivered from a factory with standards for non-allergen food production	Food prepared in the hospital kitchen
Proper communication and information distributing: Writing a procedure for food preparation and delivery to patients with food allergies	Dept. of Nutrition and Diet	Director of the Dept of Nutrition and Diet, the hospital procedures writing manager	A written procedure for food preparation and delivery to patients with food allergies, to inform all hospital staff	–
Proper communication and information dissemination: Training program	Hospital kitchen, wards and deliverers to wards	Physicians, clinical dietitians, nurses	Hospital staff and employees’ training program	–

*Dept* department

Table 2 presents the allergen ingredients in available dietary feeding formulas. Table 3 presents the foods that are restricted at hospital meals for each of the main food allergies. These materials were assimilated into the computerized hospital medical system and are available to the medical staff, hospital kitchen and food service staff.

**Table 2:** Allergen ingredients in specific dietary formula supplements

Dietary Formula	Lactose	Cow milk protein	Soy	Eggs	Fish and seafood	Wheat, gluten, flour	Nuts, ground-nuts	Tree nuts	Sesame
***Adult’s formulas***									
Osmolyte		V	V						
Jevity		V	V						
Jevity Plus		V	V			V			
Nutren 2		V	V						
^a^ Nephro HP		V	V						
Perative		V	V						
^a^ Pulmucare		V	V						
^a^ Peptamen prebio		V	V						
^a^ Ensure plus		V	V						
^a^ Easydrink		V	V						
^c^ Hadassa-shake	V	V	V	V		V	V	V	V
^c^ Thickner plus	V	V	V	V		V	V	V	V
^a^ Abound									
^a^ Module IBD		V	V						
***Children’s formulas***									
^c^ Materna RTF	V	V	V		V				
Materna Premature	V	V							
Materna sensitive	V	V	V						
Materna plant base			V						
^c^ Nutramigen									
Pregestamil									
^c^ Monogen		V						V	
Ketokal		V	V						
^c^ Neocate									
^c^ Enfamil AR	V	V							
^c^ Infatrini	V	V	V		V				
^a^ Nutren junior		V	V						
^a^ Peptamen junior		V	V						
^a^ Module IBD		V	V						
Renastart		V	V						
^c^ Similac 60/40	V	V	V						
Foramino									

V = The specific ingredient is present in the dietary formula

^a^ contains sucrose; ^b^ contains fructose; ^c^ contains coconut

**Table 3:** Foods containing allergens according to hospital meal content

Type of food allergy	Foods to avoid according to meal type
*Milk/milk products*	
Breakfast	Cheese of any kind: white cheese, cottage, yellow cheese. Dairy products such as: yogurt and cream Drinks containing milk/ milk-based: cocoa, coffee with milk, milk. Any food with milk products
Lunch	In accordance with the Kosher food menu in the hospital, milk and milk products are not served at lunch
Dinner	Foods containing milk, any kind of cheese or any other milk product such as: pizza and quiche
*Celiac: gluten intolerance- wheat flour, barley, oats and rye*	
Breakfast	Regular bread of any kind, pancakes, cakes, muffins, cornflakes and other kinds of cold cereals, hot cereals such as oatmeal and semolina, cookies, pretzels, and any product that includes gluten (wheat flour), which may include salad dressings
Lunch	Food flavored with soup powder, schnitzel or breaded fish, meatballs of any kind containing breadcrumbs, ready-made meatballs (unless clearly labeled as “gluten-free”), soy sauce, couscous, pasta, wheat groats, grits
Dinner	Regular bread of any kind, muffins, quiche and vegetable pies that include gluten, pancakes, pizza, cakes, biscuits, foods containing gluten (wheat flour), energy snacks, soy sauce
*Egg*	
Breakfast	Eggs made in any way, pancakes, cakes, cookies, muffins
Lunch	Schnitzel, chicken/turkey/meatballs, breaded fish, vegetable and chicken pies
Dinner	Eggs made in any way, pancakes, cakes, cookies, any kind of pie, muffins
*Allergy to soy (soy, tofu and other soy products)*	
Breakfast	Bread, buns, cornflakes, soy lecithin (widely used in the food industry)
Lunch	Foods cooked in soy oil, vegetarian soy-based products as well as industrialized dishes such as frozen fish meatballs (may contain soy protein), soy sauce, lecithin soy, quinoa/bulgar/ potato-based meatballs
Dinner	Foods cooked in soy oil, bread, buns, cornflakes, all industrialized products that may contain soy protein such as pies and corn schnitzel and similar products, soy sauce, soy lecithin
*Sesame allergy: (sesame, tahini, halva, sesame oil)*	
Breakfast	Bread, buns, tahini, dairy, halva, salads with sesame
Lunch	Schnitzel/ fried fish with sesame coating, raw or cooked vegetables containing sesame seeds, tahini, sesame oil
Dinner	Bread, bun, tahini, halva, salad with sesame, pretzels with sesame
^a^ Allergies to fish/seafood	
Breakfast	Tuna, sardines
Lunch	Any fish
Dinner	Tuna, sardines, any fish
Allergy to nuts/ almonds (walnuts, almonds, cashews, hazelnuts, pecans, pine nuts, pistachios, other nuts	
Breakfast	Added nuts, cornflakes/granola/morning cereals containing any kind of nuts, nut spreads, fresh soy milk
Lunch	A main course with nuts or almond additions, salad containing nuts, rice or other carbohydrates cooked with nuts
Dinner	Added nuts, quiche with nuts or snacks containing nuts, cornflakes/granola/morning cereals containing any kind of nuts, nut spreads, fresh soy milk
*Peanuts allergy*	
Breakfast	Peanuts/ packaged snacks containing peanuts, peanut butter / peanut pastry
Lunch	A main course with peanuts, any peanut-containing salads
Dinner	Peanuts/ packaged snacks containing peanuts or peanut butter

^a^ seafood is irrelevant due to kosher food

### The rationale and the status of the current intervention

The increasing prevalence of anaphylaxis caused by food allergies worldwide [2, 12] requires the attention of hospital food service systems; however, standards of practice for safety protocols are scarce [9, 10, 27]. The Israeli Ministry of Health’s policy is “patient-centered”. This is expressed by a multi-disciplinary approach that ensures personalization of treatment, patient safety and accessibility; and that includes nutritional support and diet therapy. In particular, the menu is personalized, according to medical indications and personal preferences [35]. Hospital foodservice increasingly focuses on clinical outcomes associated with nutritional intake, in addition to patient satisfaction and cost savings [35]. An example of the high-risk nature of food allergy management through hospitals was demonstrated in Australia, when food allergen ingestion during hospitalization was considered to have contributed to the death of an adolescent [27]. Hospital foodservice is considered one of the most complicated of the hospitality sectors [7, 34]. Any miscommunication in this arena can result in accidental allergen exposure, as was previously reported [10]. This has heightened the realization that staff members from diverse sectors should be involved in maintaining food allergy safety, in contrast to approaches of the past that focused exclusively on the hospital kitchen. Accordingly, a strategic intervention policy of food allergy is required to coordinate the entire process of food preparation and delivery, and the knowledge and awareness of the wide range of hospital staff. The hospital sectors involved in the process include hospital management, physicians, nursing staff, nutrition professionals, hospital kitchen workers, food delivery workers and computer coding and programing staff. Improving communication between medical systems and food services is an important strategy to reduce risks. This improved communication is based in part on the utilization of computerized systems, thus reducing the need for human communication within a busy clinical environment [10]. Importantly, the broad range of hospital employees, with their diversity in professional knowledge and specialties, poses challenges [2, 11, 30, 31]. Indeed, our experience indicates that collaboration and synergy between these sectors was initially lacking. To implement changes through the computerized system and to prioritize the issue, hospital management approvals were required, and consultations between allergy specialists and heads of internal wards were much needed to ensure the safety of the established procedures. Moreover, implementing the changes in the computerized system (e.g., preventing automatic enteral feeding for a patient with food allergy, choosing the type of food allergy, separating the list of food allergens of each patient during hospitalization, etc.), required ongoing professional education programs for the various hospital staff, due to the high turnover of employees.

A lack of documented procedures raises the risk of an accidental exposure to allergens among patients with food allergies [10, 11]. Therefore, we incorporated the interventions we performed, including those that were technically implemented in the hospital foodservice, into a written institutional procedure. In our literature search, we identified only two pre-existing standards of practice for such protocols among other national hospitals or health care systems [9, 11]. Importantly, the role of the food service dietitian who was dedicated to this project was central for assimilating the interventions that were implemented at the hospital kitchen level, including various logistical and programmatic aspects of this process.

Several changes are still needed. During the inpatient setting, medical staff should ask patients questions that will distinguish food intolerances (such as lactose intolerance) from food allergy. Notably, the current procedures aim to prevent food-related anaphylaxis events, and are not aimed to prevent food intolerance, however the issue of food intolerance should be further addressed and targeted in a future intervention and expand the current one. Furthermore, a few wards of our hospital are still lacking computer communication with the hospital kitchen. Additionally, standard recipes should be established for all meal items, which should clearly identify allergens and ingredients of concern. Substitutions in menus and recipes for patients with food allergies and food intolerances should be checked with a dietitian. Moreover, similar to the policy established regarding enteral feeding, a policy should be created for patients on parenteral feeding (to prevent the automatic delivery of an unsuitable parenteral solution to patients with food allergies). Finally, patient satisfaction and the nutritional adequacy of the menu for inpatients with food allergies should be evaluated. This is in light of reports that showed nutritional inadequacy of hospital dietary provision for patients with therapeutic dietary needs including food allergies [10, 39]. Generally, miss events are not frequently reported through health care systems, especially serious miss events. Prior to the intervention, missed events of food allergy in the hospital were not noticed, though such events probably occurred. Importantly, we were able to attain details of one serious near-miss event of anaphylaxis allergic reaction due to food (milk) cross-contact in our hospital. This was the catalyst for initiating our project. Despite the low documented incidence of missed events of food allergy in our hospital, such events are potentially life threatening. Moreover, due to the increase in the number of individuals with food allergies, hospitals worldwide should expect a similar rise among patients in the future. Notably, since the change in policy at our hospital, five near-miss events have been documented. This may indicate the success of the current initiative in raising awareness to patient safety, including the reporting of near-miss events.

The strategy and policy changes to ensure patient safety in our hospital required specific resources and collaborations. All the changes were implemented by collaborations with internal sectors of the hospital, without external financial resources, and within the hospital internal budget that was dedicated to some initial changes related costs. This included the addition of a dedicated role of a kitchen dietitian and a budget for ordering lunch meals from an outsource factory. Importantly, the awareness of health care staff to food allergy phenomenon has risen only in recent years. Moreover, the immunological pathways differ substantially between drug allergy and food allergy, as the latter involves classical allergenic proteins whereas drug allergy involves large molecules that activate the immune system by a variety of mechanisms.

Of particular emphasis is the uniqueness of the awareness and responses to food allergies compared to drug allergies in the hospital setting. When a patient is admitted to the hospital, a doctor and nurse routinely ask about allergies to medications and food. If an allergy exists, it is automatically marked both through the computer system medical file and on the patient’s identity tag. However, food allergies are perceived as less dangerous than drug allergies. Thus, while medications are given in the ward only by nurses, food is given to patients in the wards by untrained staff. This highlights the necessity for specific intervention strategies for the prevention of life-threatening events that may occur due to food allergies. Additionally, while medicines are kept in a locked room, with limited access, food in general is relatively available and accessible throughout the hospital, thus increasing the risk for mistakes that could potentially resalt in tragic events. Finally, it is important to emphasize the complex process in the hospital foodservice system that is required for preparing allergy food trays, in contrast to trays for special diets. Our hospital serves food by a “personal tray”. i.e., the tray is prepared in the kitchen and delivered to the ward with a note stating the patient’s name, identification number and diet type. More than 25 special diets are served (e.g., low-salt diet, hemodialysis diet). These diets comprise more than 50% of the food trays. Thus, the kitchen staff is well-experienced and prepared for handling these diets. In contrast, food trays for allergies are significantly less required. Yet, to avoid mistakes (that can be life-threatening), greater attention and guidance are needed. As the hospital kitchen is not able to provide the level of accuracy required to ensure patient safety and to prevent food allergies, the allergy lunch dishes are delivered specifically from a well-trained outsource (i.e., a factory with an ISO standard mark). This ensures that these trays are clean of any allergy source such as eggs and soy sesame seeds. The chain of steps leading to the arrival of food to a patient with a food allergy is complex, while any mistake through a single stage can sabotage the entire process and endanger the patient’s life.

## Policy implications

Major suggestions and policy implications for the establishment of food allergy safety protocols in hospitals are as follows:

- Food allergy is a major concern to patient safety; inadequate attention to this matter can result in severe disability and even death.
- Attention to food allergies, including a health policy, should be formulated and promoted on all managerial levels, i.e., national, hospital and hospital-department.
- A specific multidisciplinary protocol should be established according to guidelines that are appropriate at national and hospital levels, and implemented in the hospital by all the teams involved in the process. The protocol should include food preparation and delivery to patients with food allergies.
- Involvement of the hospitals’ management is crucial for implementation of policy change and monitoring outcomes.
- Training on food allergy and emergency preparedness should aim to educate all caregivers and health care workers in the hospital, including those involved in clinical admission and all employees who are involved in the process of food delivery to patients (e.g., computer department, nutrition and diet department, kitchen staff, medical and nursing teams) and those who administer adrenaline, the first-line treatment for anaphylaxis. Training should ideally be repeated and reinforced regularly, due to the high turnover of staff and their various expertise.
- Safe practices and clear communication during the hospitalization of patients with food allergies should be a major focus of policy change and protocol establishment.
- Designating and designing of a special section of the kitchen for preparing allergen-free meals, a separate pickup area for allergen-free meals, and color-coded equipment for allergen-free meals are important practices needed in a hospital setting which could potentially save lives. Additionally, menus should be created with clear ingredient lists, which are updated accordingly. Importantly, computer technology should be adjusted to facilitate filtering menus depending on food items, and to create crucial food allergy alerts as needed.

## Overall conclusions

Food allergy can be life-threatening and impose a personal and public health burden. With the increase in the number of individuals with food allergies, hospitals worldwide should expect a similar rise among hospitalized patients. Considerable investment is needed by health care systems to establish appropriate strategies and policies to ensure patient safety. Our experience suggests that policy interventions for food allergy safety require collaboration between various hospital sectors and employees, as well as hospital kitchen design. Providing suitable safe in-patient feeding requires the involvement and periodic training of all medical, nursing, dietetic, kitchen staff and computer and application team. In a technological world, computerized systems delivering food to hospitalized patients must be adapted to create uniform food safety, and institutional policies should be prioritized accordingly.

## Data Availability

The materials of the current study are available from the corresponding author on reasonable request.
